# Liver Transplantation in a Patient with Acquired Dysfibrinogenemia Who Presented with Subdural Hematoma: A Case Report

**DOI:** 10.4274/tjh.2017.0045

**Published:** 2017-12-01

**Authors:** Şencan Acar, Gökhan Güngör, Murat Dayangaç, Reyhan Diz-Küçükkaya, Yaman Tokat, Murat Akyıldız

**Affiliations:** 1 İstanbul Memorial Ataşehir Hospital, Liver Transplantation Unit, İstanbul, Turkey; 2 Konya Training and Research Hospital, Department of Internal Diseases, Konya, Turkey; 3 İstanbul Bilim University Faculty of Medicine, Department of General Surgery, İstanbul, Turkey; 4 İstanbul Bilim University Faculty of Medicine, Department of Internal Medicine, Division of Hematology, İstanbul, Turkey

**Keywords:** Dysfibrinogenemia, Liver transplantation, Subdural hematoma

## To The Editor,

Fibrinogen is one of the most abundant proteins in the blood; normal levels range from 200 to 400 mg/dL. Fibrinogen is synthesized in the liver and is essential for the clotting of blood. It also binds to platelets, supports aggregation, and plays an important role in wound healing. Fibrinogen deficiencies can be caused by decreased levels (hypo- or afibrinogenemia) or defective function (dysfibrinogenemia). Dysfibrinogenemia may either be autosomal dominantly inherited or acquired and it can manifest as bleeding or thrombotic events, or in some cases both simultaneously. Situations causing acquired dysfibrinogenemia include chronic liver disease, malignancies, and autoimmune diseases. Herein, we report a liver transplant recipient with dysfibrinogenemia who presented with subdural hematoma due to liver cirrhosis.

A 41-year-old male presented to the emergency department with headache and sicchasia. Cranial computerized tomography (CT) imaging showed subdural hematoma and surgical drainage was planned. Since he had thrombocytopenia, prolonged prothrombin time (PT), and hyperbilirubinemia, he was evaluated by a gastroenterology specialist and diagnosed with decompensated liver cirrhosis. Laboratory findings are shown in Table 1. Abdominal CT imaging showed liver cirrhosis, ascites, and splenomegaly.

The neurosurgery specialist suggested conservative treatment because of the high risk of surgery for the patient who had decompensated liver cirrhosis. The patient then consulted with the hematology department. Both the patient and his family had a negative history of bleeding. The peripheral blood smear was not consistent with disseminated intravascular coagulopathy (DIC). Thrombin time (TT) was 26 s (normal range: 16-20 s), fibrinogen activity was 106 (180-350), PT was 18.7 s (9.8-12.7 s), and activated partial thromboplastin time (aPTT) was 41.7 s (27-38.8 s). Results for the mixing test (the patient’s plasma was mixed with normal pool plasma), corrected PT, aPTT, TT, and factors X, V, VIII, and IX were within normal limits. Acquired dysfibrinogenemia because of liver cirrhosis was diagnosed based on thromboelastographic findings.

Cryoprecipitate and fresh frozen plasma were administered until coagulation test results returned to normal, but the patient’s consciousness deteriorated. Subdural hematoma drainage was then performed and consciousness dramatically improved. The patient was placed on the waiting list for cadaveric liver transplantation due to decompensated liver cirrhosis. Cadaveric liver transplantation was performed 3 months later. Posttransplant follow-up coagulation tests were dramatically improved (Table 2).

Dysfibrinogenemia can be caused by posttranslational sialylation of fibrinogen, as is seen in patients with chronic liver disease, and it can present with bleeding complications. Dysfibrinogenemia can also cause thrombotic complications if defective fibrinogen molecules are resistant to plasmin cleavage. In the presented case, dysfibrinogenemia secondary to liver cirrhosis resulted in serious bleeding that was cured following liver transplantation.

In cases of portal hypertension and liver cirrhosis, defective hemostasis can occur due to a decrease in procoagulant and anticoagulant protein synthesis, a decrease in the destruction of activated coagulant factors, functionally abnormal fibrinogen synthesis, thrombocytopenia, and abnormal platelet function [1,2,3]. Abnormal fibrinogen is found in patients with diseases characterized by increased sialic acid content. However, dysfibrinogenemia may also occur in other systemic diseases such as multiple myeloma, autoimmune disorders, and malignancy, and with the usage of some medications (glucocorticoid, isotretinoin, and antileukemic agents) [4,5].

When dysfibrinogenemia is suspected, factor deficiencies and DIC should be excluded. Normal or high antigen levels and low functional activity are commonly seen in patients with dysfibrinogenemia [6].

There is no specific treatment for dysfibrinogenemia and treatment should be personalized. Fresh frozen plasma or cryoprecipitate should be given for bleeding [7]. However, antithrombotic treatment (warfarin) should be offered in cases of thrombotic complications. Acquired dysfibrinogenemia may lead to fatal bleeding in cirrhotic patients and liver transplantation is the only curative treatment in cases of that rare complication.

## Figures and Tables

**Table 1:**
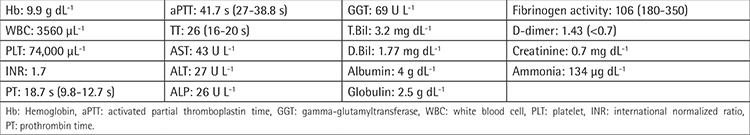
The patient’s laboratory findings.

**Table 2:**
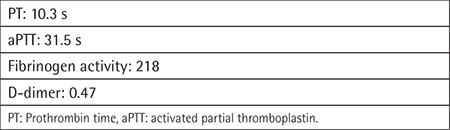
The patient’s hematological laboratory findings upon liver transplantation follow-up.
